# A novel 3DCT-based classification for posterior cruciate ligament tibial avulsion fractures

**DOI:** 10.3389/fsurg.2026.1790512

**Published:** 2026-04-01

**Authors:** Xiong Wang, Zhi Zhang, Wei Wang, Yongqing Yang, Wenqiang Wei, Shuming Zi, Liehu Cao

**Affiliations:** 1 Department of Sports Medicine, Tongji Hospital, School of Medicine, Tongji University, Shanghai, China; 2 Department of Orthopedics, Shanghai Baoshan Luodian Hospital, Baoshan District, Shanghai, China; 3 Department of Traumatic Surgery, Shanghai East Hospital, School of Medicine, Tongji University, Shanghai, China; 4 Department of Orthopedics, Shanghai Sixth People’s Hospital Affiliated to Shanghai Jiao Tong University School of Medicine, Shanghai, China; 5 Department of Orthopedics, Jinjiang Municipal Hospital, Shanghai Sixth People's Hospital Fujian, Jinjiang, Fujian, China

**Keywords:** computer tomography, meyers and mcKever, posterior cruciate ligament, three-dimensional, tibial avulsion fracture

## Abstract

**Objective:**

The Meyers and McKeever (MM) classification is widely used for posterior cruciate ligament (PCL) tibial avulsion fractures; however, it fails to comprehensively reflect fracture characteristics and morphology due to its exclusive reliance on plain radiographs, which may result in suboptimal treatment decisions. Computed tomography (CT) scanning and three-dimensional computed tomography (3DCT) reconstruction can provide a more detailed visualization of articular fracture configurations, enabling the development of effective treatment strategies. Therefore, we developed a novel classification system for PCL tibial avulsion fractures based on fracture characteristics on 3DCT images, systematically evaluated and compared classification accuracy and reliability with the MM classification.

**Methods:**

Patients aged 18 years or older who underwent plain radiographs and CT examinations that confirmed PCL tibial avulsion fractures from June 2020 to Jan 2025 were included. A novel 3DCT-based classification system was established by considering three key fracture characteristics: fracture displacement degree, fracture line numbers, and fracture involvement regions. To verify the reliability and accuracy of the novel 3DCT-based and MM classification systems, intra- and inter-rater reliability assessments were performed. Additionally, the consistency and discrepancy in fracture patterns between the two classification systems were systematically described.

**Results:**

Ultimately, 53 patients (40 males and 13 females) with PCL tibial avulsion fractures were enrolled in the final study (mean age 42.9, range 22–65). The novel 3DCT-based classification system consisted of four principles and seven categories. The intra-rater reliability of the MM classification demonstrated substantial agreement, whereas the 3DCT-based classification exhibited perfect agreement. The inter-rater reliability of both classifications displayed substantial agreement, and the novel classification had higher reliability values. In addition, approximately 22.6% of non-displaced fracture types, along with some type II and III fractures identified through radiographs, exhibited differing fracture characteristics when evaluated using 3DCT.

**Conclusion:**

The novel 3DCT-based classification is more reliable, simplified, and intuitive than the MM classification. This novel classification system allows for a more accurate description of these fractures and reduces the risk of misdiagnosis based on radiographs. Additionally, it provides valuable guidance for preoperative planning and the selection of appropriate treatment strategies.

## Background

The primary function of the posterior cruciate ligament (PCL) is to prevent posterior translation of the tibia and to maintain knee stability (1). An avulsion fracture of the tibial insertion of the PCL represents a distinct injury that can significantly impair PCL function, resulting in knee instability, cartilage degeneration, arthritis, and a diminished quality of daily life (2). PCL tibial avulsion fractures are commonly classified according to the Meyers and McKeever (MM) classification, a well-established and widely accepted system in clinical practice, which is based on fracture displacement observed on plain radiographs (3). The widespread adoption of the MM classification is primarily attributed to the simplicity and cost-effectiveness of conventional x-rays. However, the accuracy of evaluating PCL tibial avulsion fractures on x-rays may be affected by several factors, including the quality of radiography, the position of the injured knee, and the presence of overlapping femoral condyles (4). Studies have demonstrated that the precise assessment of fracture patterns and a thorough understanding of fracture characteristics are critical for guiding preoperative planning, selecting appropriate treatment strategies, and enhancing knee functional outcomes (5).

Green et al. introduced a new MRI-based classification for tibial spine fractures, which is mainly determined by the quantitative fracture fragment displacement and concomitant soft tissue impingement (6). Compared to x-ray-based classification, MRI-based classification demonstrates superior accuracy, reliability, and efficiency in identifying fractures and concomitant soft injuries (7–9). This classification provides more specific and quantitative criteria for such fracture patterns, facilitating a more nuanced understanding of treatment indications. However, its clinical application is limited by restricted availability, relatively high costs, and the potential obscuration of fracture details caused by hyperintense hemorrhage and exudates on MRI. Consequently, the development of a more convenient and effective classification system for PCL tibial avulsion fractures remains critical to directing preoperative planning and optimizing treatment strategies in clinical settings.

Currently, surgical interventions utilizing arthroscopic or open-surgical techniques, such as screws, anchors, EndoButton, hook plates, sutures, and other techniques, are recommended options with favorable outcomes for PCL tibial avulsion fractures (10–12). However, suboptimal prognoses in some cases are attributed to incomplete or inaccurate characterization of fracture patterns and inappropriate therapeutic strategies. In this context, computed tomography (CT) and three-dimensional (3D) reconstruction have emerged as indispensable examinations. These modalities excel at delineating the intricate morphological details of PCL tibial avulsion fractures, thereby enhancing diagnostic accuracy, minimizing misinterpretation, and providing a robust foundation for preoperative planning (13, 14). Moreover, they can also recognize occult fractures that are not visualized on plain radiographs (13, 15, 16).

A novel classification system named “Xu-Chen concise classification” for PCL tibial avulsion fracture was developed based on fracture morphology characteristics and posterior ligamentous complex involvement. This system enables comprehensive evaluation of fracture characteristics, facilitates optimized preoperative planning, and guides matched fixation techniques. However, the described fracture patterns are mainly confined to the posterior intercondylar fossa and fail to reflect accompanying meniscus injuries, articular surface fractures, and intercondylar damage, which may potentially compromise treatment decision-making and lead to an unfavorable prognosis (16). To overcome these limitations of the current classification system, we propose a novel fracture classification method based on the extent of fracture displacement, the number of fracture lines, and the involvement of fracture regions on 3DCT reconstruction images. The objectives of this study were to: describe the novel 3DCT-based classification system; assess its accuracy and reliability compared to the MM classification, and evaluate its value in guiding preoperative planning and treatment strategies.

## Materials and methods

### General information

A retrospective collection and analysis of consecutive patients diagnosed with PCL tibial avulsion fractures was conducted at our institution from June 2020 to Jan 2025. Basic patient information was extracted from the electronic medical records system. The Digital Imaging and Communications in Medicine (DICOM) data were retrieved from the Picture Archiving and Communication System (PACS), which included both radiographs and CT scans. For all enrolled patients, 3DCT reconstruction of the injured knee was routinely performed on UII_3D_Viewer software version 1.0 (United Imaging, Shanghai) within the imaging system. The reconstructed patella, femur, and other structures were virtually removed using the software. The posterior and superior views were selected to illustrate the detailed characteristics of the avulsion fractures. Informed consent was obtained from all patients at the time of admission, and the study was approved by the institutional review board (No. 2025-YS-080). To gain a deeper understanding of the characteristics of PCL tibial avulsion fractures, cases of anterior cruciate ligament (ACL) tibial avulsion fractures and those associated with tibial plateau fractures were excluded from the analysis. Additionally, patients presenting with open fractures, pathologic fractures, multiple fractures, historical fractures, or severe osteoarthritis were also excluded.

### The novel 3DCT-based classification system

**Figure 1:**
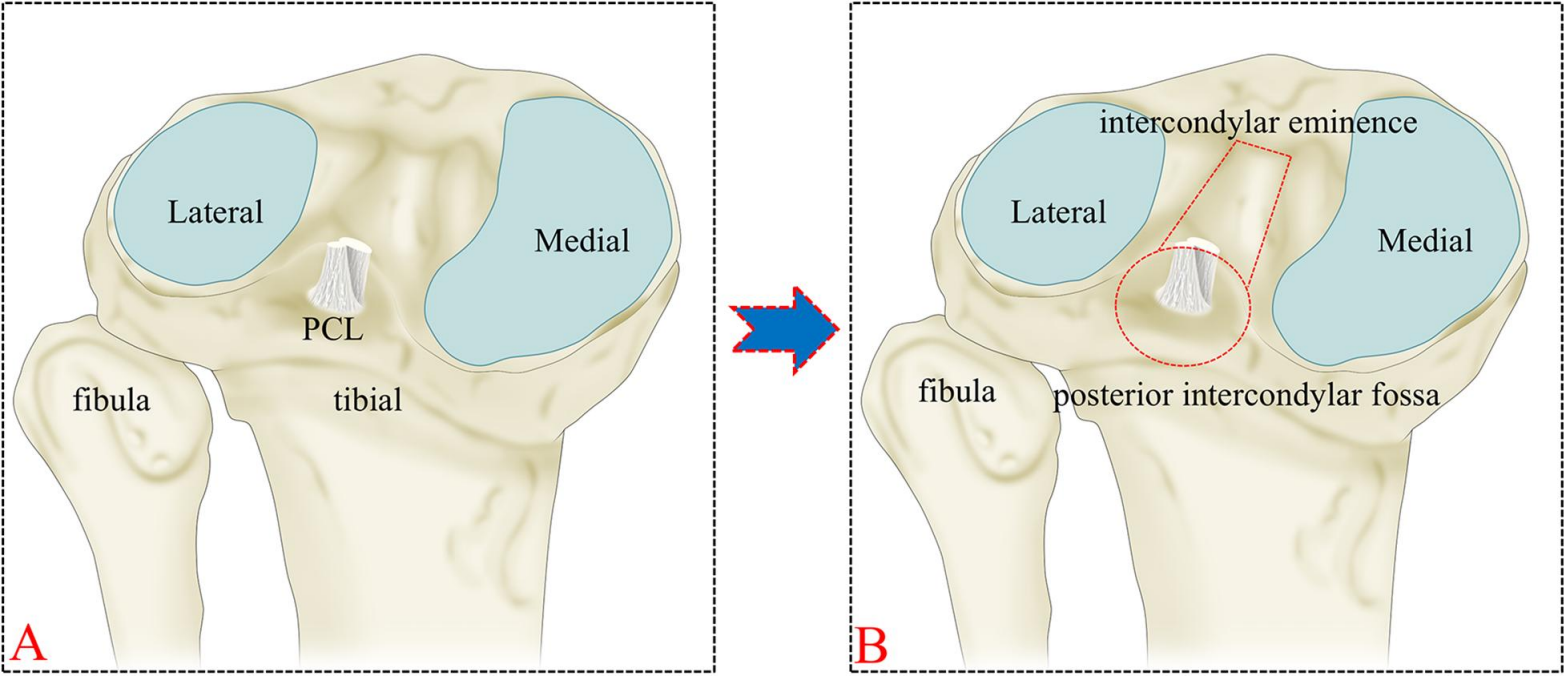
**(A)** schematic diagram of the knee joint, **(B)** regional scope corresponding to the posterior intercondylar fossa and intercondylar eminence.

**Figure 2:**
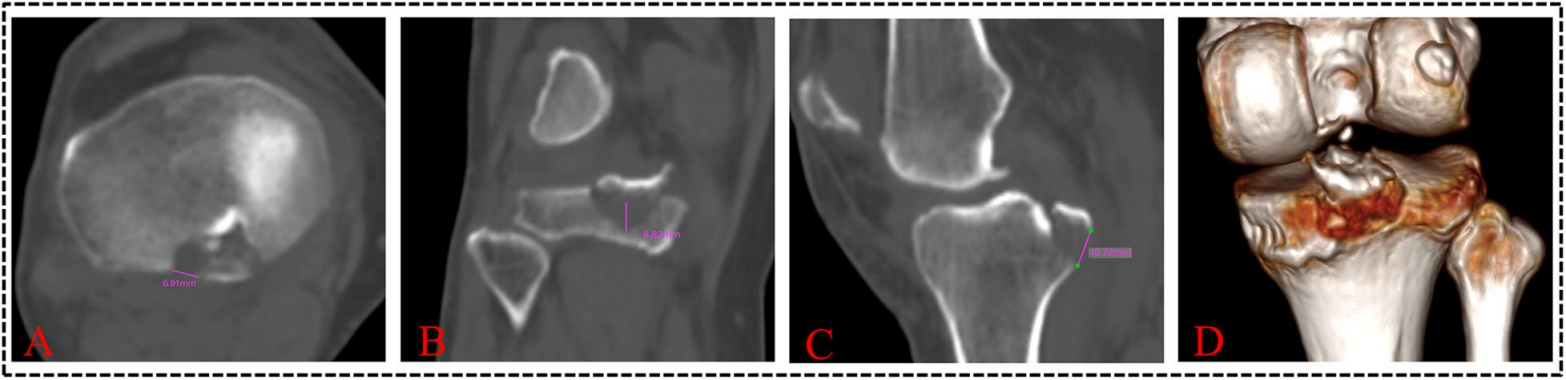
CT and 3DCT reconstruction images of PCL tibial avulsion fracture. **(A–C)** Axial, coronal, and sagittal CT planes, **(D)** 3DCT reconstructed images.

**Figure 3:**
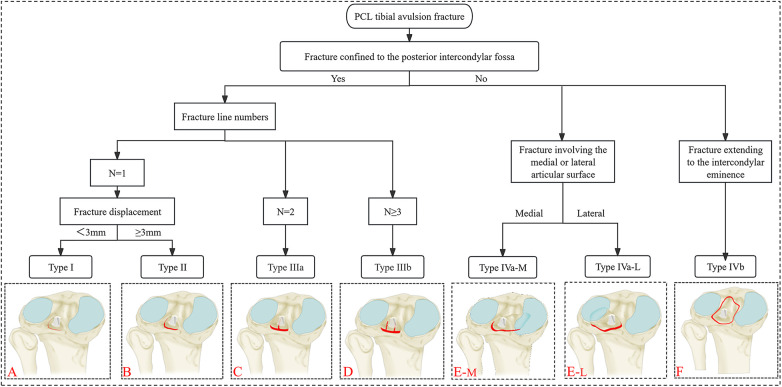
Schematic diagram for identifying characteristics and novel 3DCT-based classification of PCL tibial avulsion fractures. **(A)** Type I: Non-displaced or minimally displaced fracture (displacement <3 mm). **(B)** Type II: Displaced fracture (≥3 mm). **(C)** Type IIIa: Complex fracture with two fracture lines (*n* = 2). **(D)** Type IIIb: Comminuted fracture with a minimum of three fracture lines (*n* ≥ 3). (**E–M/L)** Type Iva-M/L: Fracture involving the medial **(M)** or lateral **(L)** articular surface. **(F)** Type IVb: Fracture extending to the intercondylar eminence.

A novel 3DCT-based classification has been introduced, focusing on the extent of fracture displacement, the number of fracture lines, and the regions of the fragments involved (confined or extended to the posterior intercondylar fossa). Figure 1 illustrates the positions and relationships of various structures within a clear knee joint model. Sagittal, coronal, and axial CT planes, along with 3DCT reconstructed images, were reviewed before further analysis (Figure 2). The sagittal plane was most frequently used for measuring fracture displacement, defined as the distance from the apex of the fracture fragment to the fracture base. Moreover, we chose 3 mm as the displacement threshold based on previous literature (16–18). A type I fracture (Figure 3A) is defined as a non-displaced or minimally displaced fracture, characterized by a displacement of less than 3 mm. A type II fracture (Figure 3B) is characterized as a displaced fracture (≥3 mm). A type IIIa (Figure 3C) is characterized by a complex fracture featuring two fracture lines. Type IIIb (Figure 3D) is characterized by a comminuted fracture exhibiting a minimum of three fracture lines. A type IV fracture is defined as a fracture that extends beyond the posterior intercondylar fossa and involves the articular surface or intercondylar eminence. Subtype IVa-M/L fracture (Figure 3E) involved the medial or lateral articular surface, while subtype IVb (Figure 3F) pertained to a fracture extending to the intercondylar eminence.

### Intra- and inter-rater reliability assessment

Three observers evaluated the imaging data of 53 cases (plain radiographs and 3DCT reconstruction images) at two different time points, using both the MM classification and novel 3DCT-based classification in random order. The evaluation interval between the two sessions was four weeks (19). The three observers were orthopedic surgeons with 13, 10, and 5 years of clinical experience, respectively. All observers were blinded to each other's assessments and were familiar with fracture classification systems. Before the evaluation, the three observers studied and calibrated the criteria of the 3DCT-based classification to ensure consistent understanding. In Session 1, the three observers independently classified all x-ray and 3D reconstruction images of the knee joint according to both the MM and 3DCT-based classifications. In Session 2, after a four-week interval, the same three observers reclassified the same set of images. All imaging data were anonymized, and the order of the images for each case was randomly arranged and numerical identifiers.

### Statistical analysis

Statistical analyses were performed using SPSS software version 25.0 (SPSS, Chicago, IL, USA). Clinical data are presented as means ± standard deviations and percentages. The objective was to analyze the reliability of the classification system as assessed by the same observer on different occasions (intra-observer reliability) and by different observers on the same occasion (inter-observer reliability). Intra- and inter-rater reliability were assessed using the weighted kappa and Fleiss’ kappa coefficients. Kappa values of less than 0 indicate no agreement; values from 0 to 0.20 indicate slight agreement, 0.21 to 0.40 indicate fair agreement, 0.41 to 0.60 indicate moderate agreement, 0.61 to 0.80 indicate substantial agreement, and 0.81 to 1.0 indicate almost perfect agreement.

## Results

A total of 53 patients (40 males and 13 females) with a mean age of 42.9 years (range from 22 to 65) were finally included. Twenty-four patients sustained injuries to the right knee, while twenty-nine patients sustained injuries to the left knee. The mechanisms of injury included two-wheeled vehicle injuries in 24 (45.3%) patients, sports-related injuries in 14 (26.4%) patients, traffic accidents in 10 (18.9%) patients, and falls from heights in 5 (9.4%) patients. Table 1 presents a summary of the demographic data and age distribution of patients with PCL tibial avulsion fractures.

**Table 1:** General information.

Variable	Value
Age, years, mean ± SD (range)	42.9 ± 10.4 (22–65)
Sex	
Male	40 (75.5%)
Female	13 (24.5%)
Laterality	
Right	24 (45.3%)
Left	29 (54.7%)
Methods of injury	
Two-wheeled vehicle injuries	24 (45.3%)
Sports-related injuries	14 (26.4%)
Traffic accidents	10 (18.9%)
Falls from heights	5 (9.4%)
Age distribution range (years)	
18–30	5 (9.4%)
30–40	15 (28.3%)
40–50	19 (35.8%)
50–60	11 (20.8%)
>60	3 (5.7%)

The intra-rater and inter-rater reliability of the 3DCT-based classification system was superior to that of the MM classification system (Table 2). The intra-rater reliability of the MM system demonstrated substantial agreement, whereas the 3DCT-based system exhibited perfect agreement. The inter-rater reliability of both the MM and 3DCT-based systems displayed substantial agreement; however, the latter had higher reliability values. In terms of intra-rater reliability, Observer 1 achieved a Kappa value of 0.619 with the MM system, compared to 0.879 with the 3DCT-based system. Observer 2 recorded a Kappa value of 0.687 with the MM system and 0.862 with the 3DCT-based system. Observer 3 showed a Kappa value of 0.650 with the MM system and 0.831 with the 3DCT-based system. The inter-rater reliability results for the first session indicated a Kappa value of 0.671 for the MM system and 0.711 for the 3DCT-based system. In contrast, the second session evaluation yielded a Kappa value of 0.635 for the MM system and 0.778 for the 3DCT-based system.

**Table 2:** Intra-rater and inter-rater reliability of the MM classification and 3DCT-based classification systems.

Observers	Meyers and McKeeve's system	3DCT-based system
Observer 1 (Intra-rater reliability)	0.619 95% CI (0.442–0.797)	0.879 95% CI (0.818–0.939)
Observer 2 (Intra-rater reliability)	0.687 95% CI (0.528–0.845)	0.862 95% CI (0.782–0.942)
Observer 3 (Intra-rater reliability)	0.650 95% CI (0.485–0.815)	0.831 95% CI (0.749–0.914)
Session 1 (Inter-rater reliability)	0.671 95% CI (0.557–0.786)	0.711 95% CI (0.636–0.787)
Session 2 (Inter-rater reliability)	0.635 95% CI (0.515–0.755)	0.778 95% CI (0.703–0.854)

**Figure 4:**
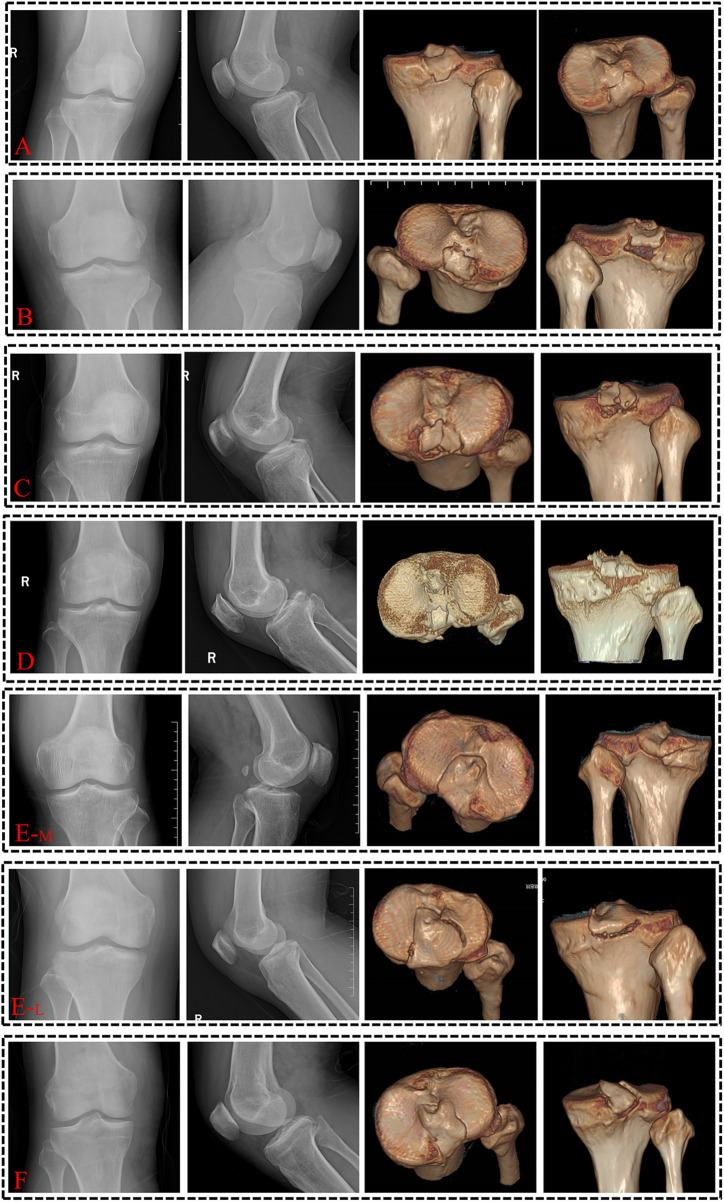
Comparative analysis of PCL tibial avulsion fracture classification between the MM system and the new 3DCT-based system. **(A)** Both the MM and 3DCT-based systems classify the fracture as Type I. **(B)** The MM system classifies the fracture as Type I, while the 3DCT-based system designates it as Type II. **(C)** The MM system classifies the fracture as Type II, while the 3DCT-based system classifies it as Type IIIa. **(D)** The MM system classifies the fracture as Type III, while the 3DCT-based system classifies it as Type IIIb. (**E–M/L**) The MM system classifies the fracture as Type I, whereas the 3DCT-based system classifies it as Type IVa-M/L fracture [involving the medial **(M)** and lateral **(L)** articular surface]. **(F)** The MM system classifies the fracture as Type I, while the 3DCT-based system classifies it as Type IVb.

Figure 4 illustrates the fracture pattern features visualized on plain radiographs and 3DCT reconstruction images. In accordance with the MM classification, 17 cases were classified as type I, 29 as type II, and 7 as type III. Conversely, using the novel 3DCT-based classification, 5 cases were categorized as type I, 18 cases as type II, 14 cases as type III (including 9 cases of type IIIa and 4 cases of type IIIb), and 17 cases as type IV (comprising 11 cases of type IVa and 6 cases of type IVb). All PCL tibial avulsion fractures were stratified according to the two classifications described above. If a controversy arises during classification, an additional observer is assigned to evaluate and determine a final decision. Notably, approximately 22.6% (12 cases) of type I non-displaced fractures initially diagnosed by radiography, along with some type II and III fractures, exhibited different fracture characteristics on 3DCT reconstruction that were easily overlooked on radiographs.

## Discussion

The most significant finding of the current study is that the novel 3DCT-based classification provides quantitative and multi-dimensional guidance for fracture classification, which is primarily based on fracture displacement, fracture lines, and the fracture-involved regions. This system facilitates the comprehensive characterization of fracture morphology and elucidates indications for appropriate treatment strategies, thereby addressing the qualitative limitation of traditional x-ray classification systems. A prior study comparing MRI-based and MM classifications reported fair to moderate intra-rater reliability and substantial inter-rater reliability (6). Our results demonstrated that the 3DCT-based classification achieved superior intra-rater and inter-rater reliability with perfect and substantial kappa values, indicating that it is more reliable than the radiograph-dependent MM classification for PCL tibial avulsion fractures.

Our results revealed that PCL tibial avulsion fractures predominantly occur in 30–60-year-old males (the prime working population), often resulting from two-wheeled vehicle accidents, traffic accidents, and sports-related trauma. Thus, appropriate treatment guided by accurate fracture characterization is critical for preserving knee function, restoring daily working capacity, and alleviating the socioeconomic burden. However, the tibial insertion of the PCL is located posterior on the tibia, making its fracture prone to underdiagnosis on plain radiographs due to bone overlap in standard anteroposterior projections, even in lateral views for minimally displaced fractures (3). The MM classification, relying solely on fracture displacement on x-ray, fails to effectively characterize fracture size, location, and involvement range, hampering its utility in preoperative planning and clinical decision-making. By contrast, 3DCT reconstructions can effectively overcome these inherent shortcomings by visualizing complex fracture morphology obscured by plain radiography, such as bone overlap or suboptimal imaging angles (15).

**Figure 5:**
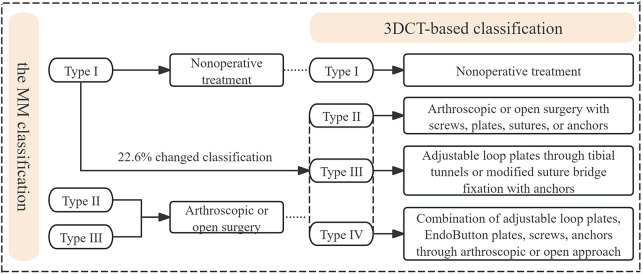
The figure chart reveals the relationship between the MM classification and 3DCT-based classification in terms of treatment strategies.

A novel “Xu-Chen concise classification” and corresponding fixation technique were described for PCL tibial avulsion fractures (16). This classification primarily focused on the fracture characteristics of the posterior intercondylar fossa and the integrity of the posterior ligamentous complex. However, some fracture cases usually extend to the articular surface and intercondylar eminence, accompanied by meniscus injuries and anterior cruciate ligament tears in the clinic. The “Xu-Chen” classification system may not effectively provide sufficient information for these fracture patterns, which may potentially affect decision-making and lead to serious consequences. Joshi et al. discovered that a high rate of meniscal and ligamentous damage is associated with tibial avulsion of the posterior cruciate ligament (TAPCL) injuries with MRI examinations and arthroscopy case records (20). They reported that one-third of TAPCLs could not be classified using the MM classification system and have meniscus or other ligament injuries. However, high cost, limited medical resources, and prolonged examination times restricted the widespread application of MRI in many clinical settings. To overcome these limitations and improve upon existing classification systems, we propose a novel 3DCT-based classification for PCL tibial avulsion fractures, according to the characteristics of avulsion fracture displacement, the number of fracture lines, and fracture region involvement. This classification is intuitive, visual, and convenient for clinicians, which can decrease misdiagnosis and provide evidence-based guidance and information for treatment strategies (Figure 5).

In clinic practice, nonoperative treatment is recommended for Type I fractures (characterized as non-displaced or minimally displaced) according to both the novel 3DCT-based and the MM classification systems (21). However, approximately 22.6% of radiographically diagnosed type I fractures exhibited occult severe fracture characteristics on 3DCT, indicating that conservative treatment might be inadequate in these cases. This finding aligns with result of Shimberg et al., who reported that 20% of MM type I fractures were associated with concomitant intra-articular injuries that necessitated surgical management (22). Therefore, 3DCT is essential for patients with suspected PCL tibial avulsion fractures on radiographs to avoid misdiagnosis and the implementation of inappropriate nonoperative management.

Surgical intervention is recommended for MM type II and III fractures (10–12, 23). Nevertheless, treatment strategies based solely on radiographs may be suboptimal due to the limited morphological information. In contrast, the 3DCT-based classification enables the formulation of precise treatment strategies to minimize postoperative complications. For type II fractures as defined by the novel 3DCT-based classification, both arthroscopic and open surgical fixation using screws, plates, sutures, or anchors is feasible (24, 25). However, the fragment size, location, bone density, and accompanying injuries must be carefully considered to minimize risks of iatrogenic fractures, intercondylar fossa impingement, or fixation failures (1, 26). Type III fractures, defined as complex or comminuted fragments with multiple fracture lines, require specialized fixation techniques, as traditional screw or plate fixation may fail to provide adequate stability. Adjustable loop plates through tibial tunnels or modified suture bridge fixation with anchors have been proposed as effective treatment methods for these challenging fractures (27–30). Additionally, maintaining comminuted fractures as a relatively intact structure is critical to ensure stable fixation. Type IV fractures are frequently overlooked or misdiagnosed on plain radiographs (9). These fractures involve the articular surface (subtype IVa-M/L: medial or lateral) and intercondylar eminence (subtype IVb), are often associated with surrounding soft tissue injuries, including meniscus compression, meniscus tears, and ligament injuries (7). Untreated concomitant soft tissue injuries can compromise knee stability and lead to a poor prognosis. Due to the extension of fragments to articular surfaces and intercondylar eminence, a combination of multiple surgical modalities is often required, such as adjustable loop plates, EndoButton plates, screws, or anchors (31). For these fracture patterns, prioritizing stable fixation of avulsion fractures combined with management of concomitant soft tissue injury, via either arthroscopic or open approach, is essential for optimizing clinical outcomes (32).

The following section outlines the limitations of the current study. First, the relatively small sample size may compromise the generalizability of the proposed classification criteria, thereby limiting their application to broader clinical populations. Second, the predominance of male patients and the exclusive inclusion of patients who underwent CT scans may introduce selection bias, as those with non-CT-diagnosed fractures were excluded. This may affect the accuracy of the novel 3DCT-based classification system in real-world applications. Third, this study did not explore potential correlations between the novel classification and injury mechanisms, gender, or age demographics, which are essential for interpreting the heterogeneity of PCL tibial avulsion fractures.

Future prospective multicenter studies with larger and more balanced cohorts are warranted to validate the reliability and further improve the generalizability of the novel classification. Furthermore, incorporation of long-term follow-up data regarding clinical outcomes and functional recovery will facilitate evaluation of the clinical value of the 3DCT-based system.

## Conclusion

The novel 3DCT-based classification system, comprising four principles and seven categories, demonstrates superior intra-rater and inter-rater reliability compared to the MM classification. It provides precise morphological characterization and valuable information about PCL tibial avulsion fractures, enabling clinicians to reduce diagnosis errors, facilitate preoperative planning, and implement effective treatment strategies.

## Data Availability

The raw data supporting the conclusions of this article will be made available by the authors, without undue reservation.
